# Chessboard Corner Detection Based on EDLines Algorithm

**DOI:** 10.3390/s22093398

**Published:** 2022-04-28

**Authors:** Xizuo Dan, Qicheng Gong, Mei Zhang, Tao Li, Guihua Li, Yonghong Wang

**Affiliations:** 1 School of Electrical Engineering and Automation, Anhui University, Hefei 230601, China; 20005@ahu.edu.cn (X.D.); z20201038@stu.ahu.edu.cn (Q.G.); hfren@126.com (M.Z.); ltaizp@163.com (T.L.); 2 School of Instrument Science and Opto-Electronics Engineering, Hefei University of Technology, Hefei 230009, China; yhwang@hfut.edu.cn

**Keywords:** corner detection, chessboard, EDLines, camera calibration, reprojection error

## Abstract

To improve the robustness and accuracy of the corner-detection algorithm, this paper proposes a camera-calibration method based on the EDLines algorithm for the automatic detection of chessboard corners. The EDLines algorithm is initially used to perform straight-line detection on the calibration image. The features of the broken straight lines at the corners are then used to filter the straight lines and remove the background straight lines outside the chessboard. The pixels in the rectangular area around the filtered straight line are sorted by the gray gradient. After using the sorted results to fit the straight line, the coordinates of the intersection of the straight lines are taken as the initial coordinates of the corners and perform subpixel optimization on them. Finally, the corner points are sorted by the conversion between pixel-coordinate systems. The camera exposure time changes and complex imaging-background experiments show that the algorithm has no missed detection and redundancy in corner detection. The average reprojection error is found to be less than 0.05 pixels, which can be used in actual calibration.

## 1. Introduction

Machine vision is extensively used in 3D measurement, 3D reconstruction, visual navigation, and target recognition [1,2,3,4]. Among them, camera calibration [5,6,7,8] is the key content in the field of machine vision. Different camera-calibration methods have been proposed, and planar 2D calibration boards made of different calibration patterns such as chessboard [9], circle [10], and concentric circles [11] are extensively used because of their convenient operation. In particular, the chessboard-calibration board is widely used in camera calibration because of its high precision and easy fabrication. The key to calibration is how to accurately detect the corner coordinates from the calibration image. At present, the corner-detection methods for chessboards primarily include gray-based [12,13,14,15,16,17] and geometric-feature-based [18,19,20,21,22,23,24,25] detection methods.

The gray-based methods primarily use the grayscale information around the corners. They are generally improved on the basis of the traditional Harris [26], SUSAN [27], and Hessian [28] corner-detection methods. For example, Teng et al. [12] used three typical local features at the corners to screen the Harris corner-detection results and identify the real corners of the chessboard. Liu et al. [13] proposed an automatic corner-detection algorithm. In the detection results of the Hessian corner detector, the algorithm uses the geometric characteristics of the corners to eliminate false corners. However, detection accuracy stays only at pixel level. Zhang et al. [14] used the detection results of the Harris algorithm and adjusted the parameters to optimize the corners. The hyperbolic tangent model of the point, using the optimal model to remove the false angle, improves the accuracy of corner detection. Xiao et al. [15] combined the Harris algorithm with the circle boundary. They used the Harris algorithm to detect corner points and the circle boundary to screen these points. Zhuo et al. [16] used the Hessian matrix to detect corner points followed by the second-order Taylor expansion to locate the subpixel coordinates at these points after obtaining the pixel-level coordinates. This strategy has a certain accuracy, but when the chessboard is rotated, the algorithm may fail. Zhu et al. [17] used an improved SUSAN corner detector for a chessboard and used the gray-gradient method to optimize the corner coordinates.

Geometric feature-based methods primarily use edge, line, and symmetry information for corner detection. Escalera et al. [18] applied Hough transform to corner detection. After using Hough transform to obtain the intersection, corners were detected around the intersection with good accuracy. Li et al. [19] used the results of the line segment detector (LSD) to filter straight lines and extract corner points. They used the gray-gradient method to optimize the corner coordinates. However, when the length of the background straight line and the chessboard straight line was close, the algorithm fails. Chen et al. [20] applied a morphological filter to extract the sub-pixel-level coordinates of the edge for polynomial fitting and found the intersection of the edge as the corner. Yang et al. [21] used Hough transform to calculate the coordinates of the intersection of chessboard straight lines as intersection coordinates and used circular template to iteratively optimize it. However, when the inclination and spacing of the background straight lines are close to the straight lines inside the chessboard, the straight lines screening effect may be reduced. Wang et al. [22,23,24] calculated the vanishing points of four straight lines around the centroid, used the vanishing points to establish the intersection point set of straight lines as the corner candidate point set, and screened the corner point set. This method will cause repeated corner calculation and increase the elimination workload. Yang et al. [25] used the surface model to fit the edge of the chessboard and extracted the intersection of the edge as the corner. When the edge is distorted and bent by the lens, the accuracy of this method will be reduced.

The present study aims to address the insufficient robustness or unverified robustness in the above methods. A corner-detection method based on the EDLines algorithm is proposed, which can improve the robustness and convenience of the algorithm while ensuring high accuracy of the detection results. The second part of this paper introduces the EDLines algorithm and the principle of the corner-detection algorithm in detail. The third part verifies the performance of the proposed method through experiments. The fourth part summarizes our work.

## 2. Chessboard Corner Detection Based on EDLines

### 2.1. Introduction to the EDlines Algorithm

The EDLines algorithm was proposed by Cuneyt Akinlar and Cihan Topal in 2011 [29], and its main function is to detect straight lines existing in an image. Compared with traditional straight-line detection algorithms such as Hough [30] and LSD [31], the EDLines algorithm detects straight lines with high accuracy and fast speed, and its straight-line detection speed is about 10 times that of the LSD algorithm.

The EDLines algorithm detects the straight lines process as follows.

The EDLines algorithm uses the edge drawing (ED) [32] edge detector to generate a set of clean and continuous edge pixel chains, namely edge segments, for a given grayscale image. The straight lines are extracted from the edge segments according to the principle of least squares. Finally, the Helmholtz principle is used to determine whether to accept or reject the extracted straight line by calculating the number of false alarms.

**Figure 1:**
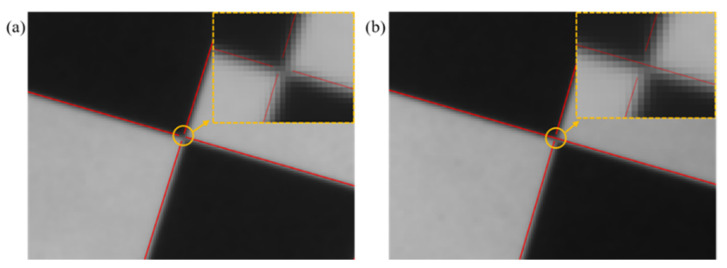
Effect drawing of straight-line fracture at corner: (**a**) breaking into four straight lines at the corner, (**b**) breaking into three straight lines at the corner.

Similar to the LSD algorithm, the EDLines algorithm can belong to only one straight line at most at each point on the image during operation. Thus, when the EDLines algorithm is used to detect chessboard straight lines, the straight line at the corner point is broken into two situations: the straight line at the corner breaks into four straight lines, as shown in Figure 1a; or the straight line at the corner breaks into three straight lines, as shown in Figure 1b.

### 2.2. Corner-Extraction Algorithm Based on EDLines

As shown in Figure 1, EDLines breaks into multiple straight lines at the corners during straight-line detection. The following methods are used to extract the corner coordinates. The specific process is as follows.

1. The EDLines algorithm is used to detect chessboard straight lines.
2. According to the different breaking conditions of the straight line at the corner point, the straight line is screened by the characteristic conditions of the broken straight line and its surrounding straight lines. The screening conditions are as follows:
  (1) When the straight line at the corner points breaks into four straight lines, the criterion is as follows:
    ① Each straight line should have three other straight-line endpoints $P_{j}$ in the neighborhood not greater than *r* from its endpoint $P_{i}$, where *i* and *j* represent the *i* and *j*th endpoints of the four straight-line endpoints.
      (1) $${\|P_{i},P_{j}\|}_{2}<r\begin{array}{cc} & i,j=1,\ldots ,4 \end{array},i\neq j$$
    ② The length of the four straight lines li should be greater than *L*, and *L* represents the shortest length of the straight line.
      (2) $$l_{i}>L\begin{array}{cc} & i=1,\ldots ,4 \end{array}$$
    ③ Among the four straight lines, two sets of straight lines are kept parallel within the angular deviation threshold $\theta_{thr}$, and their inclination angles $\theta_{i}$, $\theta_{j}$, $\theta_{k}$, and $\theta_{l}$ should satisfy the following:
      (3) $$\left\{ \begin{array}{l} ||\theta_{i}|-|\theta_{j}||<\theta_{thr}\\ ||\theta_{k}|-|\theta_{l}||<\theta_{thr} \end{array}\right.\begin{array}{cc} & i,j,k,l=1,\ldots ,4,i\neq j\neq k\neq l \end{array}$$
  (2) When the straight line at the corner points breaks into three straight lines, the criterion is as follows:
    ① Each straight line should have another straight-line endpoint $P_{j}$ within the neighborhood not greater than *r* from the endpoint $P_{i}$.
      (4) $${\|P_{i},P_{j}\|}_{2}<r\begin{array}{cc} & i,j=1,2 \end{array},i\neq j$$
    ② The distance *d* from point $P_{i}$ and point $P_{j}$ to each straight line is calculated, and the set of straight lines $\left\{S_{n}\right\}$ satisfying d<r is obtained, n=1,2,… The set of straight lines is traversed to find a straight line that satisfies the *x* or *y* coordinates of points $P_{i}$ and $P_{j}$ within the interval of its endpoints. The straight line is the third straight line in the neighborhood of the corner point, and the straight line where $P_{i}$ and $P_{j}$ are located is within the angle-deviation threshold. To maintain parallelism within $\theta_{thr}$, the inclination angles $\theta_{i}$ and $\theta_{j}$ should satisfy the following:
      (5) $$\begin{array}{cc} ||\theta_{i}|-|\theta_{j}||<\theta_{thr} & i,j=1,2 \end{array},i\neq j$$
    ③ The shortest length of each of the three straight lines li should be greater than *L*.
      (6) $$l_{i}>L\begin{array}{cc} & i=1,2,3 \end{array}$$
3. According to the above two cases, the straight lines not belonging to the chessboard are eliminated.
4. The gray gradients of pixels are sorted in a rectangular area with width *m* near the filtered straight line. The top *n* points with the largest gray gradient for least square line fitting are selected. The coordinates of the intersections of the straight lines are used as the initial coordinates of the corners.
5. The initial coordinates of the corners are optimized by the gray-gradient method, and their subpixel coordinates are obtained.
6. The transformation between pixel-coordinate systems is used to sort the corner points. The extra corner points generated by the straight lines that were not culled in step 3 are culled for subsequent camera calibration.

#### 2.2.1. Straight-Line Detection and Screening

**Figure 2:**
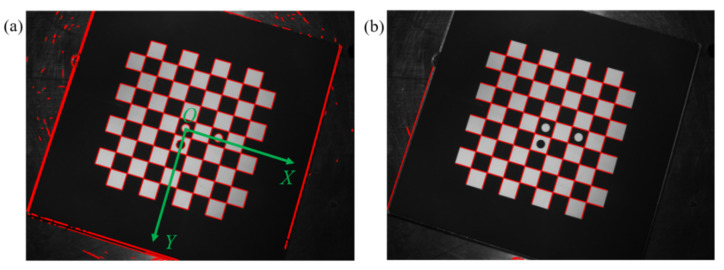
Comparison of results before and after straight-line screening: (**a**) EDLines test results, (**b**) straight-line filter results.

The conventional chessboard-calibration board is used in this experiment. The three marked circles in the center of the calibration plate are used to define the coordinate direction of the calibration plate. The two white circles are in the *x* direction, and the white and black circles are in the *y* direction, as shown in Figure 2a.

After using the EDLines algorithm to detect the chessboard-calibration plate image, the straight-line detection results as shown in Figure 2a can be obtained, including the straight lines constituting the chessboard, the background straight lines, and the straight lines introduced by noise. To eliminate the straight lines that do not constitute the corner points of the chessboard, the straight lines are screened according to the two process situations proposed in Section 2.2. Several experiments have shown that when the radius *r* of the circular domain and the length of the straight-line *L* take 10 pixels, and the angle threshold $\theta_{thr}$ takes 10°, the effect of straight-line screening is the best. The straight-line screening result is shown in Figure 2b. All straight lines around the corners of the chessboard are retained, and most of the straight lines outside the chessboard are eliminated. Only a few straight lines that have not been eliminated are retained. This part of the straight line fits false corners and is eliminated in the subsequent corner sorting.

#### 2.2.2. Corner Initial Coordinate Acquisition

**Figure 3:**
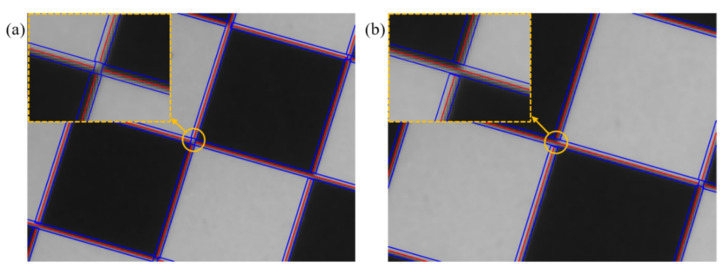
Delineation result of rectangular area around the fracture straight line: (**a**) rectangular area around the four straight lines, (**b**) rectangular area around the three straight lines.

On the basis of Figure 2b, a rectangular area with width h and the length of the straight line is delineated around each straight line. Many experiments have shown that when the value of *h* is 4 pixels in length, the calculation amount is the smallest without reducing the accuracy. When the straight line at the corner is broken into four straight lines, the result of the rectangular area delineation is shown in Figure 3a. When the straight line at the corner point is broken into three straight lines, the result of the rectangular area delineation is shown in Figure 3b.

The top *n* points with the largest gray gradient in each rectangular area are selected to perform least-square straight-line fitting. When *n* is 20, with continued increase in the *n* value, the accuracy does not change significantly. To reduce the amount of calculation, *n* is set to 20. The coordinates of the intersection of the fitted straight lines are the initial subpixel coordinates of the corners.

#### 2.2.3. Subpixel-Coordinate Optimization of Corners

Owing to the randomness of straight-line breaks at corner points, when the image quality is poor, the initial coordinates of individual corner points may be biased, which is not convenient for subsequent camera calibration, so it needs to be optimized.

The gray-gradient method is used to optimize the initial coordinates of the corner points. The principle is as follows.

**Figure 4:**
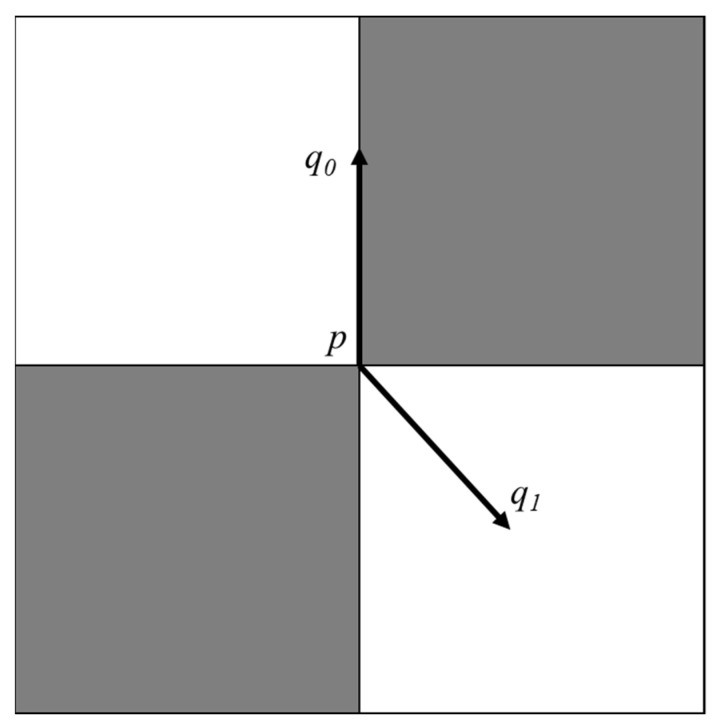
Gray-gradient image at corner.

Within the neighborhood of a corner *p*, two types of points $q_{0}$ and $q_{1}$ exist. $q_{0}$ is the point located on the edge line, and $q_{1}$ is the point located on the non-edge line in the flat area, as shown in Figure 4.

When the gray-gradient vector at point $q_{0}$ is perpendicular to the edge line:

(7) $$\vec{\nabla p}\cdot \left(q_{0}-p\right)=0$$

When the point $q_{1}$ is located in the flat area, its grayscale change is 0; that is, the grayscale-gradient vector at the point $q_{1}$ is equal to 0, so

(8) $$\vec{\nabla p}\cdot \left(q_{1}-p\right)=0$$

Among them, $\vec{\nabla p}$ represents the gray-gradient vector at the corner point *p*, $q_{0}-p$ represents the gray-gradient vector at the point $q_{0}$, and $q_{1}-p$ represents the gray-gradient vector at the point $q_{1}$. Equations (7) and (8) are true only under ideal conditions. In practice, owing to the influences of noise, illumination, lens distortion, etc., an optimization problem arises between the corner point *p* and the points in its neighborhood *N*(*p*), i.e.,

(9) $$p=\min{\sum_{q_{i}\in N\left(p\right)}\left(g_{q_{i}}^{T}\left(q_{i}-p\right)\right)}^{2}$$

Among them, *N* represents the neighborhood around corner *p*,$q_{i}$ represents any point in the neighborhood, and $g_{q_{i}}^{}$ represents the gray-gradient vector at point $q_{i}$.

Taking the derivation of *p* in Formula (9), the analytical solution can be obtained as follows:

(10) $$p={\left(\sum_{q_{i}\in N(p)}g_{q_{i}}g_{q_{i}}^{T}\right)}^{-1}\cdot \sum_{q_{i}\in N(p)}\left(g_{q_{i}}g_{q_{i}}^{T}\right)q_{i}$$

#### 2.2.4. Corner Sorting

After the above corner-point optimization, the false corner points that have not been eliminated still exist and need to be eliminated, and the remaining corner points should be sorted. The purpose of sorting is to enable correspondence of the pixel coordinates of the corner points with the world coordinates one-to-one, which is convenient for subsequent calculations.

Compared with the method of establishing the pixel-coordinate system from the upper left corner of the image in MATLAB Toolbox [33] and OpenCV [34], the chessboard-calibration board used in this paper establishes the pixel-coordinate system from the center of the calibration board. Thus, regardless of how the calibration plate is rotated, the relative position of the corner point and the pixel-coordinate system remains unchanged. Based on this characteristic, false corners can be eliminated and sorted as follows.

1. Using the EDCircles algorithm [35], the circle in the calibration image is detected, and the coordinates of the circle center are extracted.
2. The distances from all corner points to the coordinates of each circle center are calculated. The three center coordinates with the smallest sum of distances are the center coordinates of the three circles in the center of the calibration plate, thereby eliminating the false circles outside the calibration plate.
3. All subpixel corner coordinates are transformed into a new coordinate system established by the three circle centers.
4. In the new coordinate system, the distance between each corner point to the origin is calculated and sorted in ascending order. Given that the false corners are outside the chessboard pattern, all false corners can be eliminated by selecting the nearest *m* corners, where *m* is the number of corners in the chessboard.
5. The y-coordinates of the chessboard are sorted in ascending order. The corners with the same y-coordinates are then sorted in ascending order according to the x-coordinates.

## 3. Experiment Analysis

**Figure 5:**
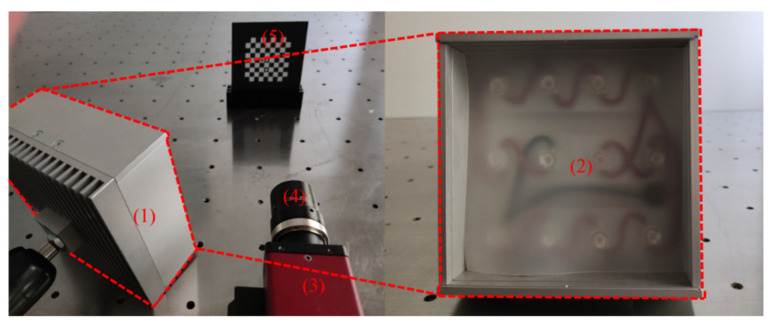
Experimental equipment diagram: (1) LED light array, (2) LED light front, (3) camera, (4) lens, and (5) calibration board.

The experimental calibration equipment is shown in Figure 5. The camera is MANTA G-201B from Allied Vision Technologies, Germany, with a maximum frame rate of 30 fps and a resolution of 1624 × 1234. The lens is Cinegon-1.4/12 from Schneider–Kreuznach, Germany. The aperture range is 1.4–11. The calibration plate is an 8 × 8 chessboard-calibration plate. The width of a single grid is 6 mm, and the diameter of the three marked circles in the center is 3 mm. The fill-light source is an LED light array with adjustable brightness.

### 3.1. Experimental Results

**Figure 6:**
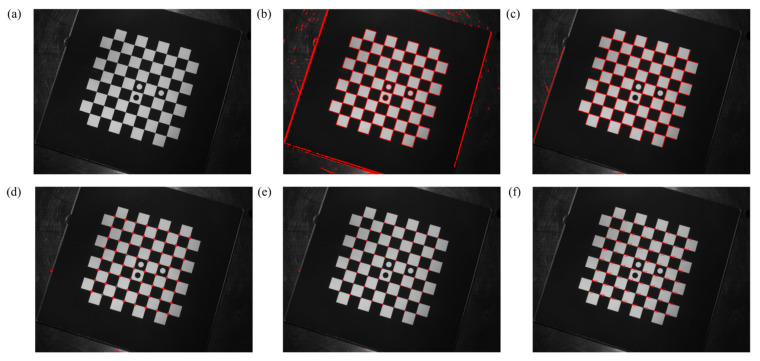
Corner-detection process: (**a**) original image, (**b**) image after straight-line detection by EDLines, (**c**) result after straight-line screening, (**d**) initial coordinate results of corner points, (**e**) result of corner points subpixel optimization, and (**f**) result after corner sorting.

A picture from normal lighting, moderate exposure, and non-complex imaging background is selected to demonstrate the process of corner detection in this paper, as shown in Figure 6.

### 3.2. Robustness Experiment

To test the robustness of the corner-detection algorithm, this study verifies it from the perspectives of exposure-time change and calibration-environment complexity. The corner-detection results area then compared with those from the literature [36] and MATLAB Toolbox. Owing to the particularity of the OpenCV corner-detection algorithm, it fails to detect corner points from the calibration plate with the characteristic circle, so this experiment is not compared.

#### 3.2.1. Robustness Verification for Overexposure and Underexposure

**Figure 7:**
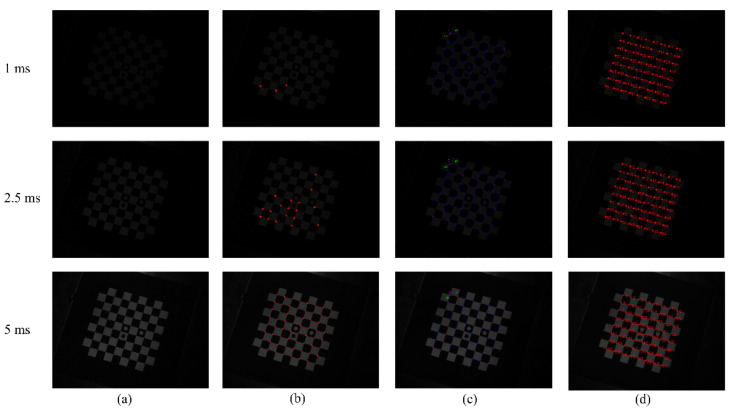
Comparison of corner-detection results in underexposure state: (**a**) original image, (**b**) corner-detection result of the literature [36] method, (**c**) corner-detection result of MATLAB Toolbox, and (**d**) corner-detection results of the method in this paper.

**Figure 8:**
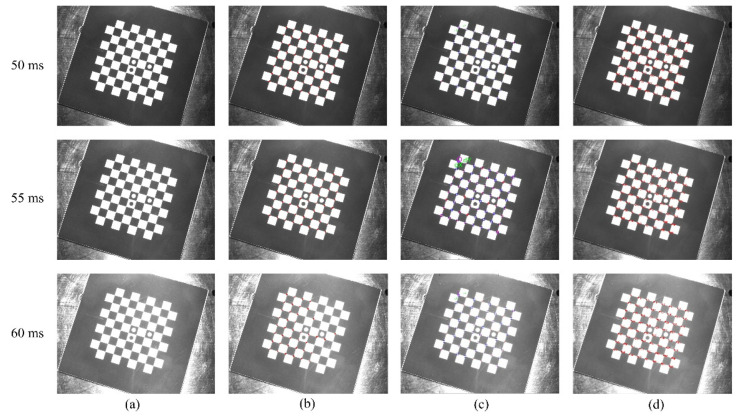
Comparison of corner-detection results in overexposure state: (**a**) original image, (**b**) corner-detection result of the literature [36] method, (**c**) corner-detection result of MATLAB Toolbox, and (**d**) corner-detection result of the method in this paper.

With other conditions unchanged, the exposure time is adjusted, and the image undergoes obvious light and dark changes. Accordingly, the corner points are extracted, and the results are compared with those of the other two methods. Considering the situation under extreme exposure time, through actual measurement, the image is completely black and the corners are undetectable when the exposure time is less than 1 ms. When the exposure time is greater than 60 ms, the image is too bright, and the black squares in the chessboard are separated. Thus, taking a comprehensive consideration, six sets of images captured at different exposure times were set up. Figure 7a is the image taken under the camera’s underexposure state, and the exposure times are 1, 2.5, and 5 ms. Figure 8a is the image taken under the camera’s overexposure state, and the exposure times are 50, 55, and 60 ms, respectively. Figure 7b–d and Figure 8b–d are the corner-detection results of the literature [36], MATLAB Toolbox, and present methods.

Under different exposure times, the number of corner points detected by the three methods is shown in Table 1.

Table 1 shows that when the literature [36] method performs corner detection, when the camera is in the underexposure state, the number of detected corners decreases with decreased exposure time. When the camera is in the overexposure state, similar to the underexposure state, the number of detected corner points gradually decreases with increased exposure time. The literature [36] method is greatly affected by the camera exposure time, and the robustness is poor when the camera is underexposed and overexposed. Using MATLAB Toolbox can always accurately detect all corner points without causing missed detection or false detection. Therefore, both the method proposed in this paper and the MATLAB toolbox have high robustness when the camera exposure time changes.

#### 3.2.2. Robustness Verification in Complex Backgrounds

**Figure 9:**
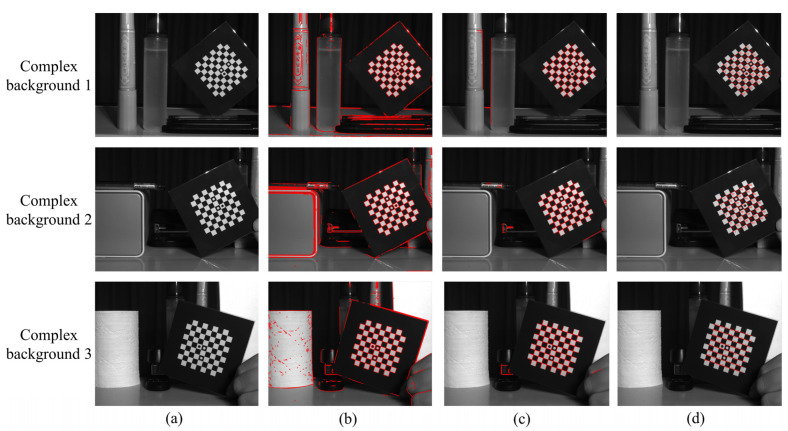
Detection of corner points under complex background: (**a**) original image before detection, (**b**)straight-line detection result, (**c**) result after straight-line screening, and (**d**) result after sorting corner points.

To verify the robustness of the algorithm’s robustness in detecting corners in complex imaging backgrounds, three groups of pictures were taken for detection, as shown in Figure 9. Figure 9a–d are the original image before detection, the result of straight-line detection, the result of straight-line screening, and the result after sorting corner points, respectively. The corresponding straight-line filter rate is shown in Table 2.

Table 2 shows that under the three complex backgrounds, after the straight-line screening in this paper, the number of irrelevant background straight lines is greatly reduced. The background straight-line filtering rate is above 90%, and the background straight lines that are not eliminated by the filtering conditions generate fewer false background corners. Thus, the workload for subsequent corner sorting is reduced. This part of the background false corners is eliminated by corner sorting, and the accurate corner-detection result is obtained, as shown in Figure 9d.

### 3.3. Convenience Comparison

Through the robustness comparison experiment in Section 3.2, the robustness of the method in this paper under the extreme exposure time of the camera is better than that of the method in literature [36], but we cannot judge which is better between our method and MATLAB toolbox from the point of view of robustness. Therefore, this section compares the convenience of the method in this paper and MATLAB toolbox to verify that the method proposed in this paper has good convenience in operation.

**Figure 10:**
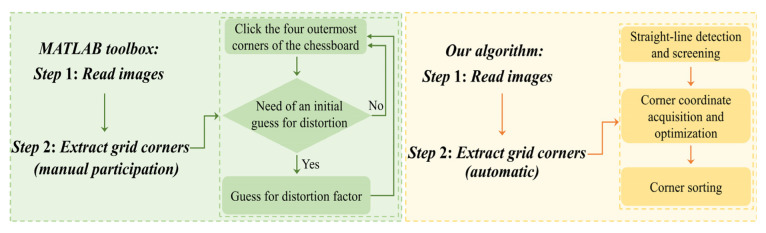
The flow chart comparison between our algorithm and MATLAB toolbox.

Figure 10 shows the comparison between the method in this paper and the flow chart of MATLAB toolbox. MATLAB toolbox needs to manually determine the four corners at the most edge of each calibrated picture in the corner extraction stage. By connecting the four edge corners into a rectangular area, the corners are extracted at the edge and inside of the rectangular area. Manual corner selection itself has large errors. When the number of images involved in calibration is large, the workload of manual operation will increase greatly and will lead to the accumulation of errors in the process of manual selection. In the corner detection stage, the method proposed in this paper automatically realizes from the line detection stage to the final corner sorting stage through the algorithm, without additional manual participation. In contrast, the method in this paper is more convenient in practical application and reduces the risk of introducing additional errors.

### 3.4. Accuracy Verification

**Figure 11:**
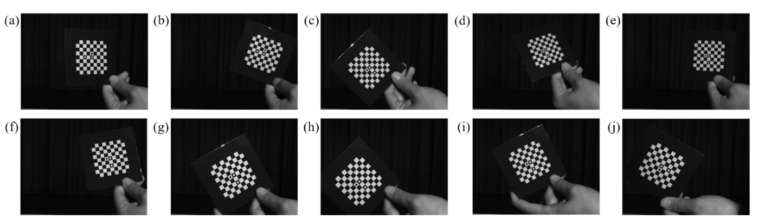
Calibration images (**a**–**j**) captured in different pose.

By calculating the reprojection error, it can be verified whether the subpixel corner coordinates obtained by the above method meet the calibration requirements. Ten captured calibration pictures are selected for the calculation of reprojection error, as shown in Figure 11a–j. When calculating the reprojection error, we need to consider the effect of lens distortion on the calculation results. Therefore, the distortion of the initial image needs to be corrected to reduce the error caused by lens distortion. The distortion correction function is defined as:

(11) $$\{\begin{array}{c} x^{\prime }=x\left(1+K_{1}r^{2}+K_{2}r^{4}\right)+2P_{1}y+P_{2}\left(r^{2}+2x^{2}\right)\\ y^{\prime }=y\left(1+K_{1}r^{2}+K_{2}r^{4}\right)+2P_{2}x+P_{1}\left(r^{2}+2y^{2}\right) \end{array}$$

**Figure 12:**
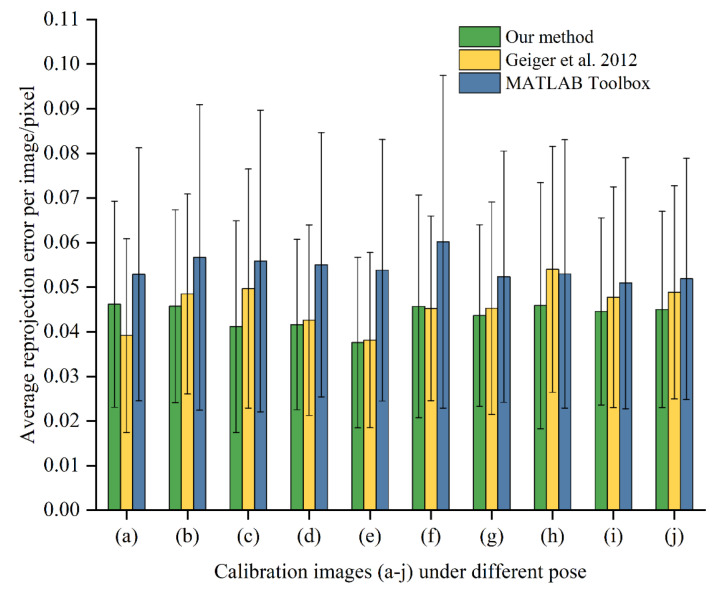
Comparison of reprojection error with literature [36] and MATLAB toolbox.

**Figure 13:**
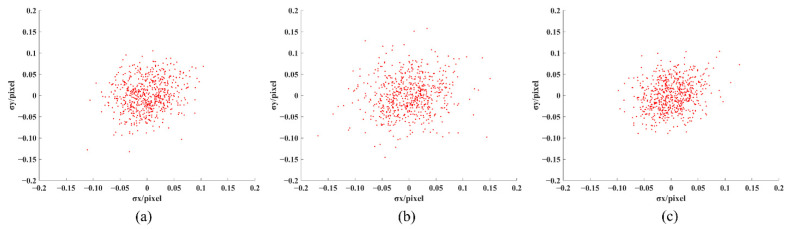
Reprojection-error scatter plot: (**a**) Literature [36] algorithm, (**b**) MATLAB Toolbox, and (**c**) Our algorithm.

Among them, *K*_1_ and *K*_2_ are radial distortion coefficients, respectively, *P*_1_ and *P*_2_ are tangential distortion coefficients, respectively, $r^{2}=x^{2}+y^{2}$, and the second-order distortion coefficient is enough to solve the conventional large distortion. After correcting the distorted image points $\left(x^{\prime },y^{\prime }\right)$, the approximate ideal image points (x,y) can be obtained. After correcting the distortion of the calibration images, the reprojection error calculation results are compared with the results of literature algorithm [36] and MATLAB Toolbox, as shown in Table 3. Among them, *f_x_* and *f_y_* are the equivalent focal lengths in the *x* and *y* directions, *μ*_0_ and *ν*_0_ are the center of the image, *s* is the tilt coefficient, and *σ* is the average reprojection error of 10 calibration images. The average reprojection-error comparison of the 64 corners of each image is shown in Figure 12. Figure 13a–c show the scatter plots of reprojection errors in the *x* and *y* directions of the 10 images obtained using the algorithm [36], MATLAB Toolbox, and present methods.

Figure 12 shows that the maximum average reprojection error of a single image of the method proposed in this paper is no more than 0.05 pixel, and the maximum standard deviation in the error bar is no more than 0.028. Figure 13 shows that the reprojection error of our method is primarily concentrated within the range of 0.1 pixel, and the maximum reprojection error in the *x* and *y* directions does not exceed 0.15 pixel. Compared with MATLAB Toolbox and the literature [36] algorithm, the reprojection error of our algorithm is more concentrated. The reprojection error of the algorithm in this paper is smaller, and the calibration accuracy can meet the calibration requirements.

## 4. Conclusions

A method of extracting chessboard corner points is proposed based on EDLines algorithm. The method uses the length and angle characteristics of the broken straight line at the corner point to screen the straight line and performs straight-line fitting on the *n* points with the largest gray gradient on both sides of the straight line after screening to obtain the initial coordinates of the corner point. Then, the gray-gradient method is used to optimize the initial coordinates to obtain the subpixel coordinates of the corner points and finally sort the corner points for actual calibration. The experiment verifies the robustness of the algorithm in this paper under complex background and camera underexposure and overexposure conditions. The result are compared with those of the literature [36] algorithm and MATLAB Toolbox. The detection results of our corner-detection algorithm are better than the algorithm in literature [36] under the conditions of camera underexposure and overexposure. Compared with the MATLAB Toolbox calibration process, the one in this paper is more convenient. In terms of calibration accuracy, the reprojection error of the proposed algorithm is smaller, which can meet actual calibration needs.

## Figures and Tables

**Table 1:** Comparison of the number of corners detected by the three methods.

Exposure Time (ms)	Literature [36] Algorithm	MATLAB Toolbox	Our Algorithm
1	3	64	64
2.5	18	64	64
5	63	64	64
50	64	64	64
55	61	64	64
60	40	64	64

**Table 2:** Background straight-line filter rate.

Complex Background	Number of Background Straight Lines before Screening	Number of Background Straight Lines after Screening	Background Straight-Line Filter Rate
Complex background 1	419	8	98.1%
Complex background 2	321	26	92.0%
Complex background 3	341	14	95.9%

**Table 3:** Camera calibration results.

Method	*f_x_*/Pixel	*f_y_*/Pixel	*μ*_0_/Pixel	*ν*_0_/Pixel	*s*	*K*_1_	*K*_2_	*P*_1_	*P*_2_	*σ*/Pixel
Our algorithm	2906.97	2907.83	802.14	632.03	0	−0.150	0.385	6.3 × 10^−4^	1.9 × 10^−4^	0.044
Literature [36] algorithm	2899.84	2900.47	803.15	628.76	0	−0.168	1.160	7.8 × 10^−4^	1.6 × 10^−4^	0.046
MATLAB Toolbox	2911.23	2911.94	802.47	632.54	0	−0.171	1.295	1.2 × 10^−3^	2.7 × 10^−4^	0.054

## Data Availability

Not applicable.
